# *NUP98::Nsd1* and *FLT3*-ITD collaborate to generate acute myeloid leukemia

**DOI:** 10.1038/s41375-023-01913-0

**Published:** 2023-05-05

**Authors:** Toshihiro Matsukawa, Mianmian Yin, Nupur Nigam, Vijay Negi, Li Li, Donald Small, Yuelin J. Zhu, Robert L. Walker, Paul S. Meltzer, Peter D. Aplan

**Affiliations:** 1 Genetics Branch, Center for Cancer Research, National Cancer Institute, National Institutes of Health, Bethesda, MD USA; 2 Department of Oncology, Johns Hopkins University School of Medicine, Baltimore, MD USA; 3 Department of Oncology & Department of Pediatrics, Johns Hopkins University School of Medicine, Baltimore, MD USA; 4 Myeloid Malignancies Program, National Institutes of Health, Bethesda, MD USA

**Keywords:** Cancer models, Cancer genetics, Haematological cancer

## To the Editor:

Acute myeloid leukemia (AML) is a heterogeneous hematologic malignancy driven largely by gene mutations and epigenetic modifications [1, 2]. The Nucleoporin 98 kDa (NUP98) gene is a component of the nuclear pore complex that also plays a role as an intranuclear transcription scaffold [3]. Fusion genes involving *NUP98* have been recognized in a wide array of hematologic malignancy, most commonly AML [4]. Among over 30 partner genes known to be fused to *NUP98* in human leukemia, *NSD1* (for Nuclear receptor-binding SET Domain protein 1) (*NSD1*) is the most common [1, 5]. Patients with *NUP98::NSD1* gene fusions have a poor prognosis, and the leukemic blasts frequently have an internal tandem duplication (ITD) of the FMS-related tyrosine kinase 3 gene (*FLT3*) gene accompanying the *NUP98::NSD1* fusion [6, 7]. Previous reports have utilized BM transduction with retroviral vectors followed by transplantation into recipient mice to model AML driven by a *NUP98::NSD1* fusion [8, 9]. In one study, most mice transduced with a *NUP98::Nsd1* fusion died of AML within 100 days [8], whereas a second study reported that mice transplanted with murine BM cells which were transduced with a *NUP98::NSD1* fusion did not develop AML but instead were euthanized due to a myeloid hyperplasia [9]. Given that genetically engineered mice offer certain advantages over retroviral transduction models, such as consistent transgene expression and integration effects, lack of ionizing radiation, and transferability between investigators, we generated *NUP98::Nsd1* transgenic mice.

**Fig. 1:**
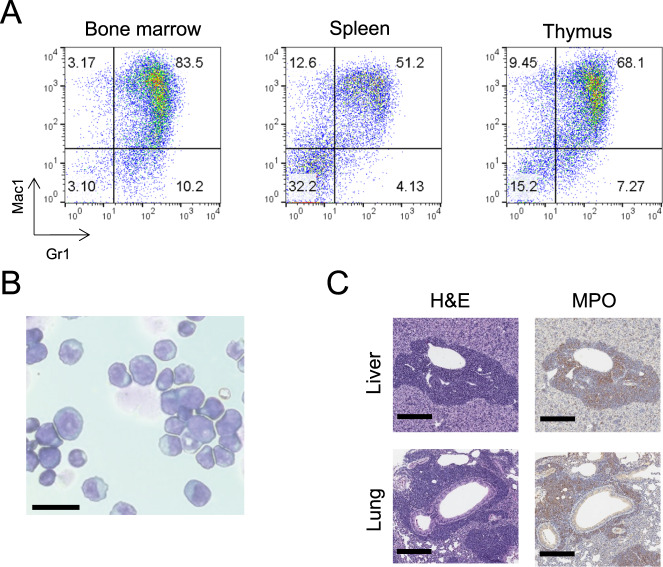
Generation of *NUP98::Nsd1* transgenic mice on a C57BL/6 background. **A** Flow cytometry demonstrates infiltration of Mac-1^+^/Gr-1^+^ cells in bone marrow, spleen, and thymus from the A10 founder. **B** Cytospin from mouse A10 BM showing myeloblasts with high nuclear/cytoplasmic ratio. Scale bar = 50 µm. **C** Invasion of lung and liver with sheets of myeloblasts positive for myeloperoxidase (MPO) Scale bar = 300 µm.

Transgenic mice that expressed a *NUP98::Nsd1* fusion gene in the hematopoietic compartment were generated by microinjection of fertilized C57BL/6 embryos (Supplementary Fig. 1A, B), as described in the Supplementary Methods. Two of seven founders (mice A10 and I8) developed leukemia during a 20-month observation period, at 181 and 232 days, respectively (Supplementary Fig. 1C). CBCs from both founders that developed AML showed leukocytosis, mild anemia, and circulating blasts (Supplementary Table S1). Flow cytometry demonstrated infiltration of bone marrow (BM), spleen, and thymus with Mac-1^+^Gr-1^+^ positive myeloblasts, and BM cytospin revealed sheets of myeloblasts (Fig. 1A, B). Necropsy findings revealed hepatosplenomegaly, and IHC demonstrated myeloperoxidase (MPO) positive blast invasion of perivascular regions of parenchymal tissues such as liver and lung (Fig. 1C). Only one of these two leukemic mice (A10) was able to successfully breed with wild-type (WT) mates prior to death from AML. Surprisingly, F1 mice from this founder did not show a survival difference between WT and transgenic mice, and only one animal (C970) in a cohort of 30 F1 transgenic mice showed evidence of AML (Supplementary Fig. S2).

To determine if there may be a mouse strain, or integration effect leading to lack of transmission of the leukemic phenotype, we generated a new cohort of transgenic mice, this time on an FVB/NJ background. In this cohort, only one mouse (MT1502) developed clear evidence of AML during a 20-month observation period. Similar to the *NUP98::Nsd1* mice generated on a C57BL/6 background, the leukemic MT1502 mouse displayed leukocytosis and anemia in the peripheral blood (Supplementary Table S1) and invasion of myeloblasts by flow cytometry, May-Grünwald-Giemsa (MG) staining, and IHC. (Supplementary Fig. S3A–C) The MT1502 founder was bred to a WT mate, but similar to findings with the C57BL/6 founder, there was no survival difference in the F1 generation between transgenic and WT F1 generation mice (Supplementary Fig. S3D).

**Fig. 2:**
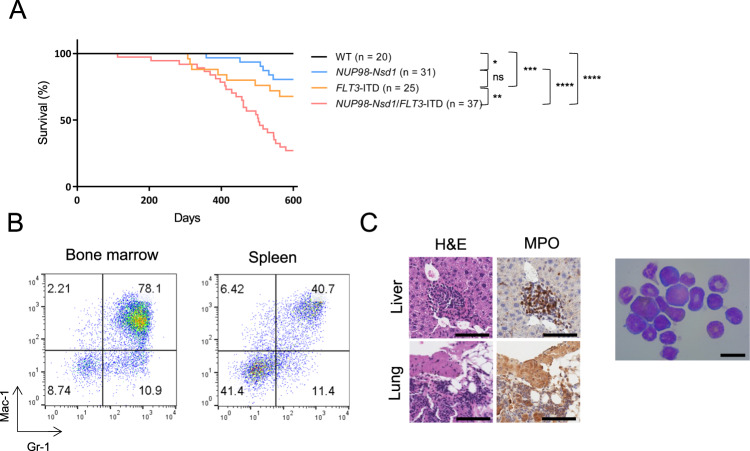
*NUP98::Nsd1*/*FLT3*-ITD double transgenic mice have decreased survival and develop AML or pre-T LBL. **A** Kaplan–Meyer curve shows decreased survival of *NUP98::Nsd1*/ *FLT3*-ITD double transgenic mice compared to WT or single transgenic mice. ns; not significant, *p < 0.05, **p < 0.01, ***p < 0.001, ****p < 0.0001. **B** Example of AML (mouse K758) with invasion of Mac-1^+^/Gr-1^+^ cells in bone marrow and spleen. **C** Invasion of myeloperoxidase (MPO)-positive myeloblasts in liver and lung. Scale bar = 100 µm (left). Myeloblasts in BM cytospin; scale bar = 20 µm (right).

Given the frequent co-occurrence of *NUP98::NSD1* and *FLT3*-ITD in human AML, we generated double transgenic mice that expressed both the *NUP98::Nsd1* fusion and a *FLT3*-ITD mutation, both on a C57BL/6 background, using FLT3-ITD “knock-in” mice as described by Li et al. [10] (see Supplementary Methods for details). The double transgenic mice (*n* = 37) had decreased survival compared to WT (*n* = 20; *p* < 0.0001), *FLT3*-ITD (*n* = 25; *p* = 0.0040), or *NUP98::Nsd1* (*n* = 31; *p* < 0.0001). (Fig. 2A) We were able to perform detailed necropsies on 17 of the deceased *NUP98::Nsd1*/*FLT3*-ITD mice. Fourteen animals (82.4%) developed AML, and three animals developed a precursor T-cell lymphoblastic leukemia/lymphoma (pre-T LBL) (Supplementary Table S2); *FLT3*-ITD only mice developed myeloproliferative disease, as previously shown [10]. The double transgenic AML were characterized by Mac-1^+^Gr-1^+^ blasts (Fig. 2B), whereas the pre-T LBL cases displayed T-lineage lymphoblasts. BM cytospin and IHC shows invasion of myeloblasts, consistent with AML (Fig. 2C). Supplementary Table S2 summarizes data from *NUP98::NSD1*/*FLT3*-ITD leukemic mice, including survival, diagnosis, relevant immunophenotype, and CBC results. Prominent, recurrent findings included severe macrocytic anemia, leukocytosis, and occasional thrombocytopenia.

We used Whole Exome Sequencing (WES) to search for acquired mutations in the *NUP98::Nsd1*/*FLT3*-ITD leukemias. Previously, we identified acquired mutations involving Ras (*Kras*, *Nras*, *Ptpn11*, *Nf1,* and *Cbl*) or tyrosine kinase (*Flt3*, *Kit*, *Jak*, *Stat*, and *Sh2b3*) genes in 20–72% of leukemias driven by NUP98 fusion proteins or the related CALM-AF10 fusion protein [11, 12]. Surprisingly, we found no recurrent acquired mutations in 14 *NUP98::Nsd1*/*FLT3*-ITD mice with AML or pre-T LBL. Rare Tier1 acquired mutations involving known leukemia genes such as *Notch1* and *Jak1* were identified in AML and pre-T LBL, respectively (Supplementary Fig. S4A, Supplementary Table S3). WES data is deposited in Sequence Read Archive (SRA), accession PRJNA952665. Given that loss of the WT allele is a frequent event in AML patients who have a *FLT3*-ITD, we evaluated *NUP98::Nsd1*/*FLT3*-ITD AML sample for loss of the WT *Flt3* allele. Four of 13 (31%) AML samples showed loss of the WT *Flt3* allele, while none of the samples lost the *FLT3-ITD* nor the *NUP98::Nsd1* allele (Supplementary Fig. S4B).

We used RNA-Seq to generate gene expression profiles for *NUP98::Nsd1*/*FLT3*-ITD AML, and compared them to gene expression profiles from WT unfractionated BM and WT BM enriched for hematopoietic stem and progenitor cells (Lineage negative BM) (Supplementary Table S4); RNA-Seq data is deposited with Gene Expression Omnibus (GEO), accession number GSE229501. Principal component analysis (PCA) demonstrated clear distinction between these three groups (Supplementary Fig. S5). Unsupervised hierarchical clustering also separated the samples into anticipated groups for *NUP98::Nsd1*/*FLT3*-ITD AML, WT unfractionated BM and Lineage negative BM (Supplementary Fig. S6A). A set of genes that was >2 fold differentially expressed at *p* < 0.05 between *NUP98::Nsd1*/*FLT3*-ITD AML and Lineage negative BM, was used to interrogate the “cell type signature” sets available on Molecular Signatures Database (MSigDB) v7.5.1.4. Gene sets that had a normalized enrichment score (NER) > 1.5 are listed in Supplementary Table S5. The majority of these gene sets represent tissue macrophages or neutrophils; several examples are shown in Supplementary Fig. S6B. Given that Gene set enrichment analysis (GSEA) suggested that the *NUP98::Nsd1*/*FLT3*-ITD AML were of myelomonocytic origin, we stained *NUP98::Nsd1*/*Flt3*-ITD AML with additional antigens. All five AML analyzed showed a similar pattern, Mac-1^+^Gr-1^+^CD16/32^+^F4/80^het^CD13^-^, consistent with myelomonocytic cells (Supplementary Fig. S7). In addition, a common theme seen with numerous *NUP98* fusion genes [4], including the *NUP98::NSD1* fusion [7–9], is enforced expression of *HOXA/B* cluster genes. Consistent with these prior studies, *Hoxa/b* genes were also upregulated in AML samples from *NUP98::Nsd1*/*FLT3*-ITD mice. A proposed mechanism for the upregulation of *HOXA/B genes* is H3K36 methylation of histones at *HOXA/B* promoters via the SET domain of NSD1, which is retained in the *NUP98::Nsd1* fusion [4, 8].

GSEA comparison to previously described AML signatures revealed strong similarity to “Valk_AML_Cluster_5”, which primarily consisted of patients with a monocytic or myelomonocytic (M4 or M5) AML subtype [13], and a group of AML patients with MLL gene fusions [14] (Supplementary Fig. S8A). The similarity between the *NUP98::Nsd1/FLT3*-ITD gene expression profile and that of *MLL* gene fusions with M4/M5 AML is consistent with recent observations that NUP98 fusion proteins are dependent on interaction with MLL protein [15]. We next compared the *NUP98::Nsd1*/*FLT3*-ITD AML gene signature to that of human *NUP98::NSD1/FLT3-ITD* AML or human *FLT3-ITD*-only AML extracted from publicly available data [7]. Again, there was strong similarity between human *NUP98::NSD1* AML and murine *NUP98::Nsd1*/*FLT3*-ITD AML (Supplementary Fig. S8B).

In summary, we have generated and characterized a genetically engineered model for *NUP98::NSD1* AML, the most common leukemic *NUP98* fusion seen in AML patients. We find that the penetrance of *NUP98::Nsd1* fusion is relatively low, and is increased dramatically by the addition of a *FLT3*-ITD, in keeping with the observation that 80% of human *NUP98::NSD1* patients also have a *FLT3*-ITD [6, 7]. The paucity of acquired somatic mutations detected by WES suggests that co-expression of *NUP98::Nsd1* and *FLT3*-ITD may be largely sufficient for the generation of AML, albeit with a variable and extended latency. A number of additional similarities can be seen between the *NUP98::Nsd1/FLT3*-ITD model characterized here and human patients with *NUP98::NSD1* fusion and *FLT3*-ITD, including immunophenotype, gene expression profiles, and loss of the WT copy of *Flt3*. These similarities suggest that the murine *NUP98::Nsd1/FLT3*-ITD genetic model described here reliably recapitulates the human disease.

## Supplementary information

Supplemental Methods

Supplemental Figures

Supp Table 1

Supp Table 2

Supp Table 3

Supp Table 4

Supp Table 5

## Data Availability

Whole Exome Sequencing (WES) is available from SRA (accession PRJNA952665). RNA-Seq data is available from GEO (accession number GSE229501).
