# Supplementary material for: NUP98::Nsd1 and FLT3-ITD collaborate to generate acute myeloid leukemia
Source: Leukemia. 2023 May 5;37(7):1545–8. doi: 10.1038/s41375-023-01913-0 (PMC10317830; doi:10.1038/s41375-023-01913-0)
Supplement: Supplementary file 1 — Supplemental Methods [file 41375_2023_1913_MOESM1_ESM.docx]

**Supplemental Methods:**

**Generation of Transgenic *NUP98::Nsd1* Mice**

The *NUP98::NSD1* fusion construct, which is a human-murine chimera used in prior studies of NUP98::Nsd1 fusion proteins, was initially generated by Wang et al^1^ and was cloned into a Vav1 expression vector, as previously described.^2^ The purified construct was injected in fertilized embryos (either C57BL/6 or FVB/NJ) as previously described.^2^ *FLT3*-ITD mice with an 18 bp ITD insertion isolated from a patient with AML “knocked-into” the murine Flt3 locus were generated as previously reported.^3^ Genotyping was performed as previously described, using primers *NUP98::Nsd1* (*NUP98::Nsd1*-geno1Fw: 5’- AACCTAAGATTGGAGGGCCTCTT-3’, and *NUP98::Nsd1*-geno2Rw: 5’- GCCTCTCGTTCAAGAACTGGAG-3’) and *FLT3*-ITD (mITD-5F: 5’- CTCTCGGGAACTCCCACTTA-3’, and mITD-3R: 5’- TGCAGATGATCCAGGTGACT-3’) with 1% and 3.5% gels, respectively. Animal experiments were approved by the National Cancer Institute Intramural Animal Care and Use Committee.

**Complete blood count (CBC), Flow Cytometry (FCM), and Immunohistochemistry (IHC)**

Serial CBCs were analyzed on peripheral blood using a HEMAVET Multispecies Hematology Analyzer (CDC Technologies). Hematologic malignancies were evaluated using published guidelines ^4^. FCM was performed as described previously ^5^ with the following antibodies: Mac-1 (CD11b)-PE (Clone-M1/70; eBioscience), Ly-6G/Ly-6C (Gr-1)-FITC (Clone-RB6-8C5; eBioscience), CD4-PE (Clone-GK1.5; eBioscience), CD8a-FITC (Clone-53-6.7; BD Biosciences), CD45R/B220-FITC (Clone-RA3-6B2; BD Biosciences), CD19-PE (Clone-1D3; eBioscience), Ter119-FITC (Clone-TER119; Biolegend), CD71-PE (Clone-R17217; Biolegend) , Ly-6A/E (Sca-1)-PE (Clone-D7; eBioscience), CD117 (c-Kit)-FITC (Clone-2B8; eBioscience). IHC was also performed as previously described ^5^. Briefly, formalin-fixed paraffin-embedded tissue sections were stained with hematoxylin and eosin (H&E), CD3 (Clone-CD3-12; Bio-Rad), Ter119 (Clone-TER119; BioLegend), and myeloperoxidase (Cat#A0398; Dako). Stained sections were scanned with Aperio AT2 digital slide scanner (Leica Biosystems), and the images were observed with Aperio ImageScope software (Leica Biosystems).

**Gene expression analysis**

RNA samples were prepared using TRIzol reagent (Invitrogen) following the manufacturer’s recommended protocol. The TruSeq Stranded Total RNA Library Prep kit from Illumina was used for library preparation. Ribosomal RNA (rRNA) was removed using biotinylated, target-specific oligos and Ribo-Zero rRNA removal beads. RNA was fragmented and first strand cDNA generated using reverse transcriptase and random primers. Double-strand cDNA was used as the input for Illumina library prep and the final purified library was sequenced on a NovaSeq for paired end (2x101 cycles) sequencing.

Alignment was performed using “STAR” aligner and quantification was done using “Quantify to annotation model” (Partek® Flow® software, v10.0). Normalization was performed using “Poscounts (Deseq2 only)” method. Low expressing genes were filtered by removing features/genes with geometric mean of 10 or lower for all samples. Gene-specific analysis (GSA) statistical modeling approach was used to test for differential expression of genes using Partek Flow.

Gene set enrichment analysis (GSEA) was performed using GSEA software (v 4.2.3). Ranked gene lists (with P value<=0.05 and two-fold differential expression) were generated, using Partek Flow. The ranked gene list was used for enrichment analysis of annotated gene sets available on Molecular Signatures Database (MSigDB) v7.5.1, including “Hallmark Gene Sets” and “Cell Type Signature Gene Sets”.

A gene set comparing genes >2-fold upregulated in *NUP98::NSD1/FLT3-ITD* AML patients (n=9) to non-*NUP98::NSD1* AML patients (n=146), and a gene set comparing genes >2-fold upregulated in *FLT3-ITD* only AML patients (n=28) to non-*NUP98::NSD1,* non-*FLT3* AML patients (n=118) was generated from GSE178855. ^6^ AML with driver mutations similar to *NUP98* fusions (*MLL*-rearranged, *NPM1* mutant) were excluded from analysis. These genesets were used for GSEA comparison to genes upregulated >2-fold in NUP98::Nsd1/FLT3-ITD mouse AML compared to mouse lineage negative (LN) bone marrow RNA.

**Whole-Exome Sequencing (WES)**

Data processing and variant calling procedure followed the Best Practices workflow recommended by the Broad Institute. The raw sequencing reads were mapped to mouse genome build 10 (mm10) by the Burrows-Wheeler Aligner followed by local realignment using the GATK suite from the Broad Institute, and duplicated the Picard tools marked reads.

Somatic variants were first filtered with the GATK recommended filtering criteria and further filtered by the following criteria: (1). Minimum fraction of altered reads 0.3; (2) Minimum number of altered reads in a tumor >= 2. (3) Log transformed FISHER p-value >= 1.5. (4) Variant impact annotation from SnpEff effect prediction is ‘High’ or ‘Moderate’ (5) Exclude SNPs reported in dbSNP build 137 or previously identified as germline variants in the WT NIH C57BL/6 or FVB/NJ colony.

**Statistics**

Data are reported as mean values ±­_­_ standard deviation, and analyzed using the Mantel-Cox log-rank test, unpaired Students’ t-test. GraphPad Prism software ver 8.4.3 (GraphPad Software, LLC) carried out all statistical analyses in this manuscript.

**References**

1. Wang GG, Cai L, Pasillas MP, Kamps MP. NUP98-NSD1 links H3K36 methylation to Hox-A gene activation and leukaemogenesis. *Nat Cell Biol* 2007 Jul; **9**(7)**:** 804-812.

2. Gough SM, Lee F, Yang F, Walker RL, Zhu YJ, Pineda M*, et al.* NUP98-PHF23 is a chromatin-modifying oncoprotein that causes a wide array of leukemias sensitive to inhibition of PHD histone reader function. *Cancer Discov* 2014 May; **4**(5)**:** 564-577.

3. Li L, Piloto O, Nguyen HB, Greenberg K, Takamiya K, Racke F*, et al.* Knock-in of an internal tandem duplication mutation into murine FLT3 confers myeloproliferative disease in a mouse model. *Blood* 2008 Apr 1; **111**(7)**:** 3849-3858.

4. Kogan SC, Ward JM, Anver MR, Berman JJ, Brayton C, Cardiff RD*, et al.* Bethesda proposals for classification of nonlymphoid hematopoietic neoplasms in mice. *Blood* 2002 Jul 1; **100**(1)**:** 238-245.

5. Gough SM, Slape CI, Aplan PD. NUP98 gene fusions and hematopoietic malignancies: common themes and new biologic insights. *Blood* 2011 Dec 8; **118**(24)**:** 6247-6257.

6. Hollink IH, van den Heuvel-Eibrink MM, Arentsen-Peters ST, Pratcorona M, Abbas S, Kuipers JE*, et al.* NUP98/NSD1 characterizes a novel poor prognostic group in acute myeloid leukemia with a distinct HOX gene expression pattern. *Blood* 2011 Sep 29; **118**(13)**:** 3645-3656.
