# Supplementary material for: NUP98::Nsd1 and FLT3-ITD collaborate to generate acute myeloid leukemia
Source: Leukemia. 2023 May 5;37(7):1545–8. doi: 10.1038/s41375-023-01913-0 (PMC10317830; doi:10.1038/s41375-023-01913-0)
Supplement: Supplementary file 2 — Supplemental Figures [file 41375_2023_1913_MOESM2_ESM.pptx]

## Slide 1
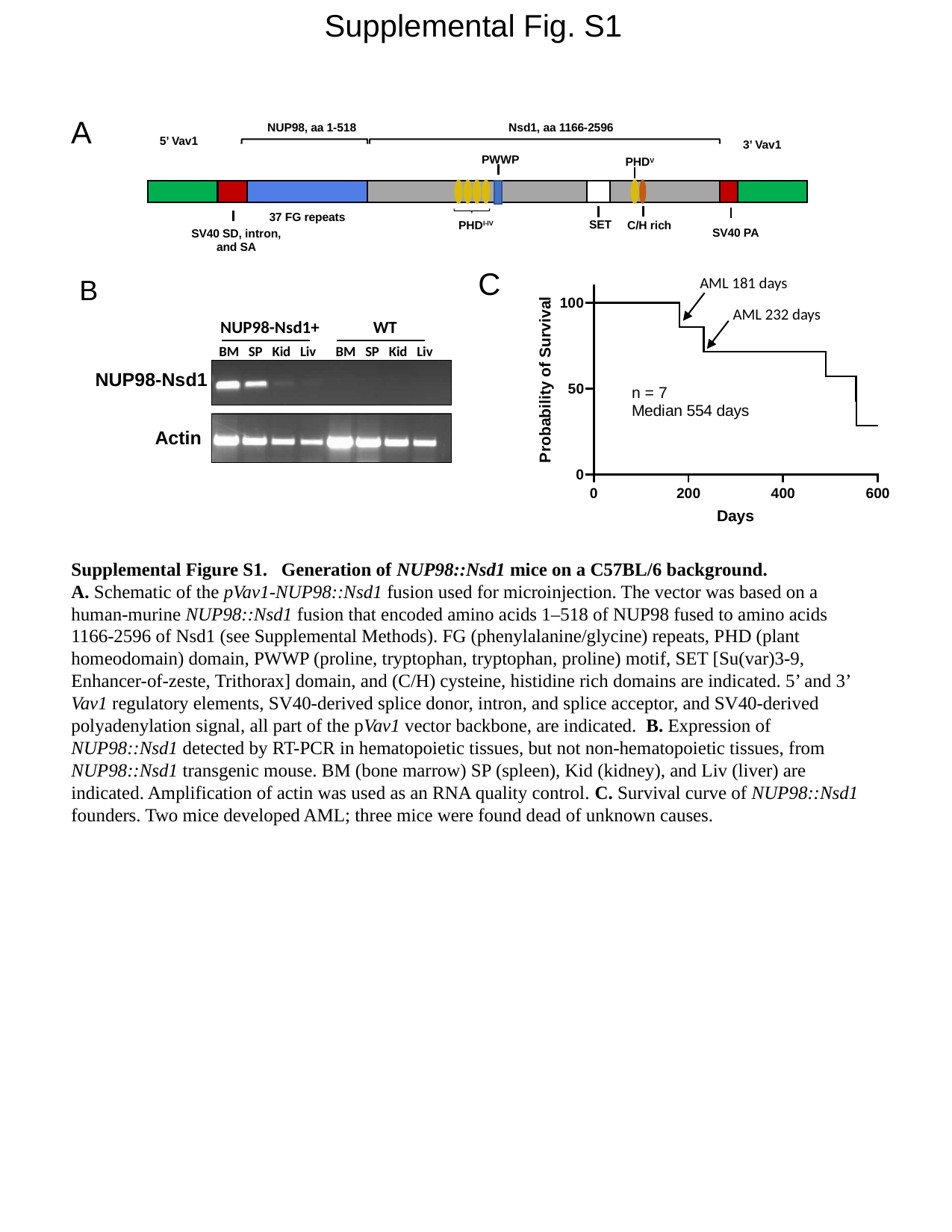

Supplemental Fig. S1
A
NUP98, aa 1-518 Nsd1, aa 1166-2596
5’ Vav1
3’ Vav1
PWWP
PHDV
37 FG repeats
SET
PHDI-IV
C/H rich
SV40 PA
SV40 SD, intron, and SA
C
B
AML 181 days
AML 232 days
NUP98-Nsd1+
WT
BM SP Kid Liv
BM SP Kid Liv
NUP98-Nsd1
Actin
Supplemental Figure S1. Generation of NUP98::Nsd1 mice on a C57BL/6 background.
A. Schematic of the pVav1-NUP98::Nsd1 fusion used for microinjection. The vector was based on a human-murine NUP98::Nsd1 fusion that encoded amino acids 1–518 of NUP98 fused to amino acids 1166-2596 of Nsd1 (see Supplemental Methods). FG (phenylalanine/glycine) repeats, PHD (plant homeodomain) domain, PWWP (proline, tryptophan, tryptophan, proline) motif, SET [Su(var)3-9, Enhancer-of-zeste, Trithorax] domain, and (C/H) cysteine, histidine rich domains are indicated. 5’ and 3’ Vav1 regulatory elements, SV40-derived splice donor, intron, and splice acceptor, and SV40-derived polyadenylation signal, all part of the pVav1 vector backbone, are indicated. B. Expression of NUP98::Nsd1 detected by RT-PCR in hematopoietic tissues, but not non-hematopoietic tissues, from NUP98::Nsd1 transgenic mouse. BM (bone marrow) SP (spleen), Kid (kidney), and Liv (liver) are indicated. Amplification of actin was used as an RNA quality control. C. Survival curve of NUP98::Nsd1 founders. Two mice developed AML; three mice were found dead of unknown causes.

## Slide 2
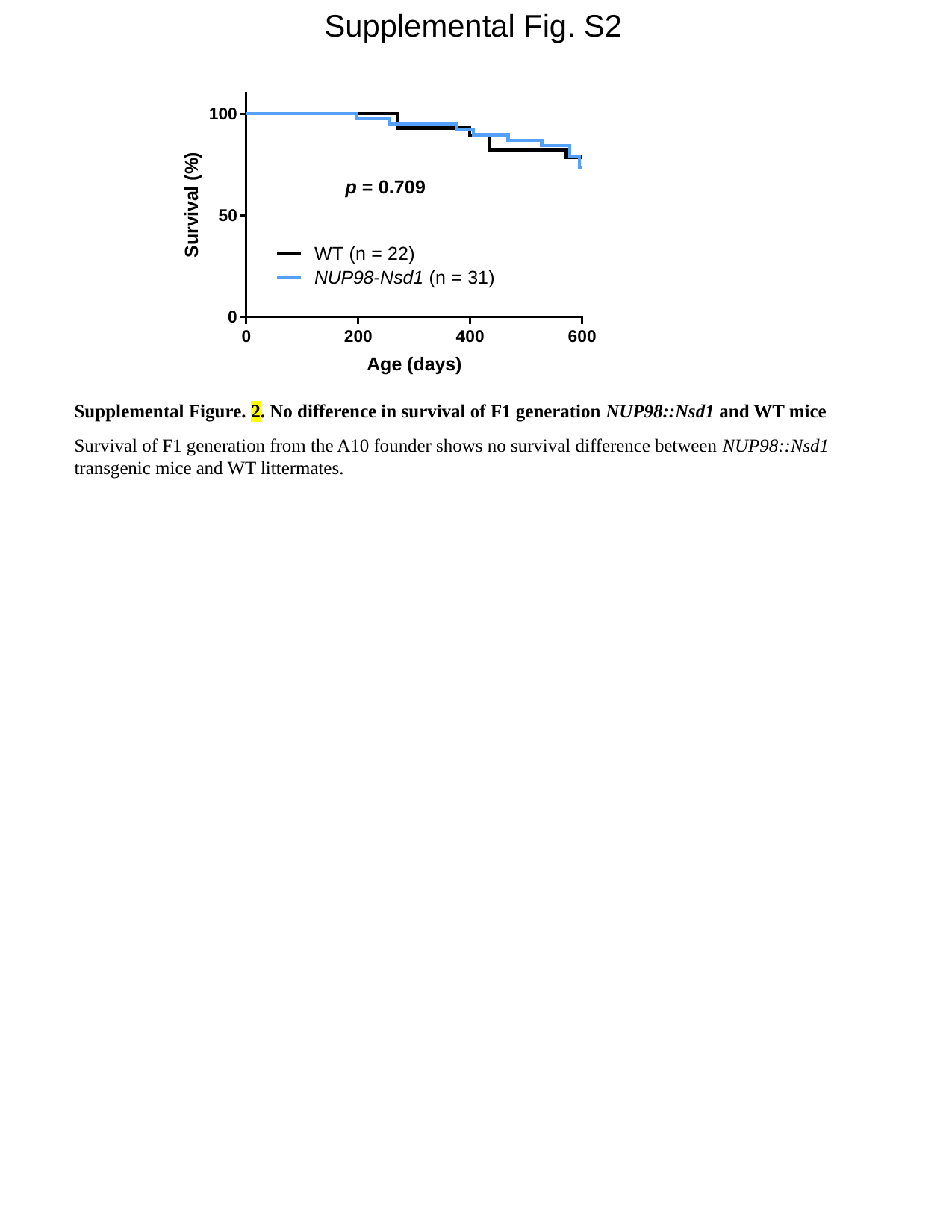

Supplemental Fig. S2
Supplemental Figure. 2. No difference in survival of F1 generation NUP98::Nsd1 and WT mice
Survival of F1 generation from the A10 founder shows no survival difference between NUP98::Nsd1 transgenic mice and WT littermates.

## Slide 3
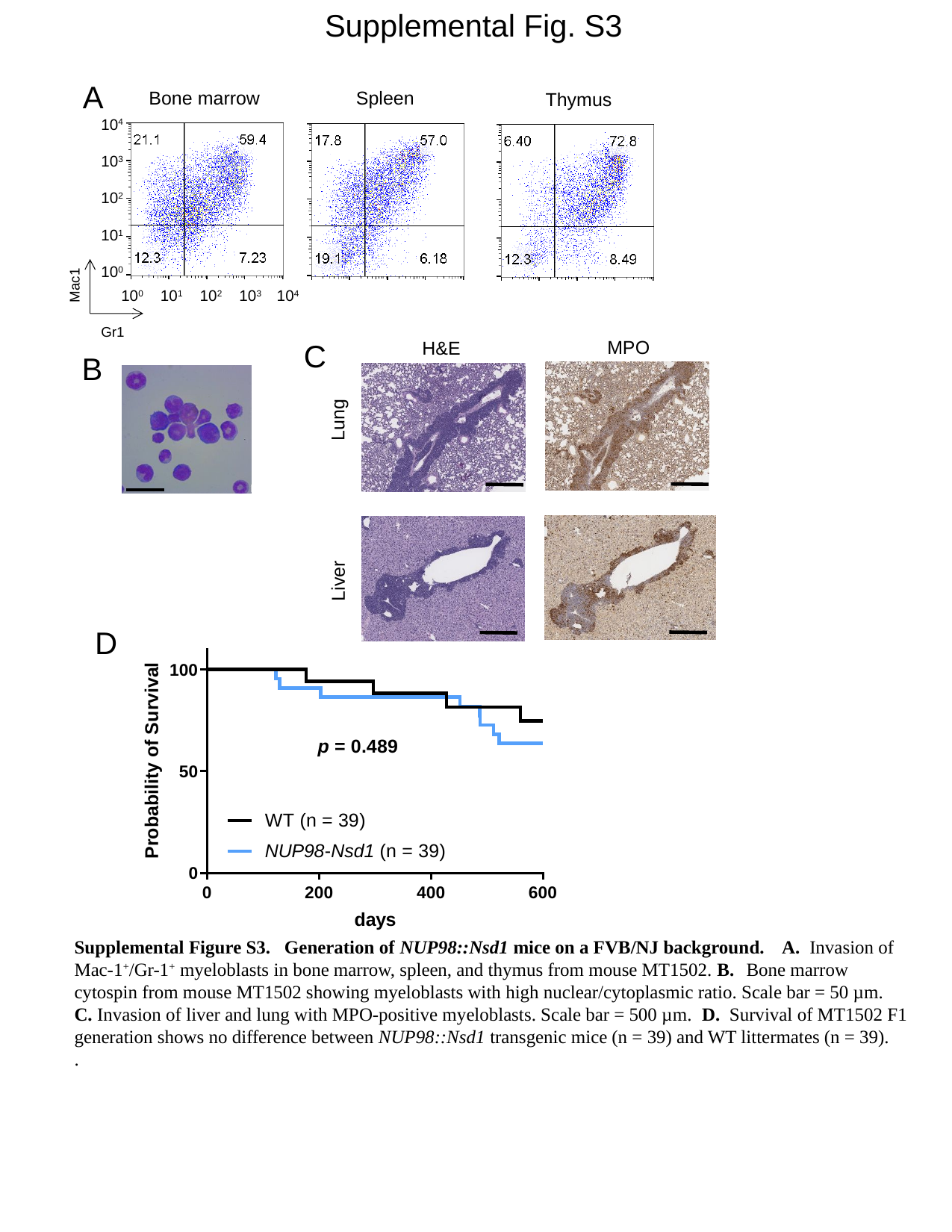

Supplemental Fig. S3
A
Bone marrow
Spleen
Thymus
104
103
102
101
100
Mac1
100 101 102 103 104
Gr1
MPO
H&E
Lung
Liver
C
B
D
Supplemental Figure S3. Generation of NUP98::Nsd1 mice on a FVB/NJ background. A. Invasion of Mac-1+/Gr-1+ myeloblasts in bone marrow, spleen, and thymus from mouse MT1502. B.	Bone marrow cytospin from mouse MT1502 showing myeloblasts with high nuclear/cytoplasmic ratio. Scale bar = 50 µm. C. Invasion of liver and lung with MPO-positive myeloblasts. Scale bar = 500 µm. D. Survival of MT1502 F1 generation shows no difference between NUP98::Nsd1 transgenic mice (n = 39) and WT littermates (n = 39).
.

## Slide 4
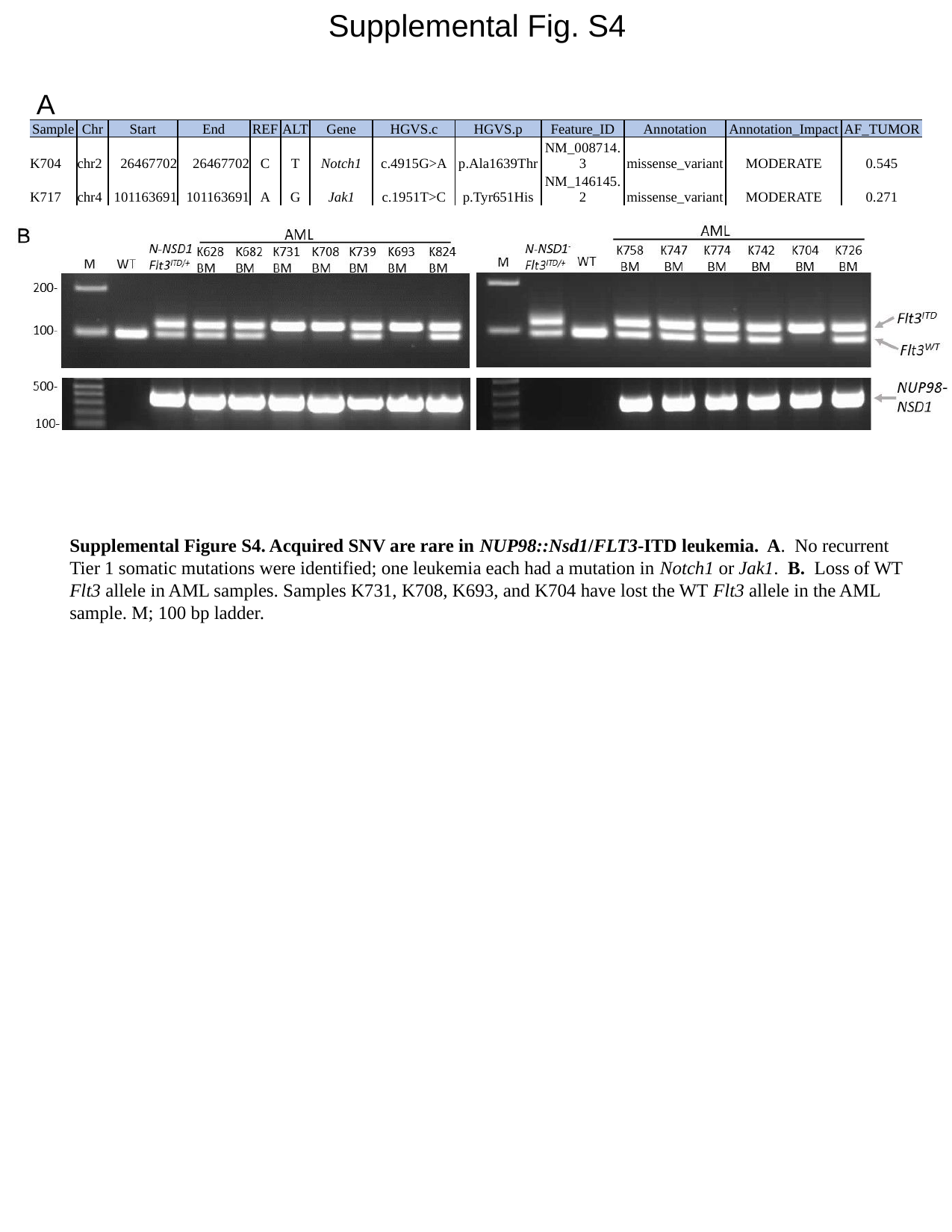

Supplemental Fig. S4
A
| Sample | Chr | Start | End | REF | ALT | Gene | HGVS.c | HGVS.p | Feature\_ID | Annotation | Annotation\_Impact | AF\_TUMOR |
| --- | --- | --- | --- | --- | --- | --- | --- | --- | --- | --- | --- | --- |
| K704 | chr2 | 26467702 | 26467702 | C | T | Notch1 | c.4915G>A | p.Ala1639Thr | NM\_008714.3 | missense\_variant | MODERATE | 0.545 |
| K717 | chr4 | 101163691 | 101163691 | A | G | Jak1 | c.1951T>C | p.Tyr651His | NM\_146145.2 | missense\_variant | MODERATE | 0.271 |
Supplemental Figure S4. Acquired SNV are rare in NUP98::Nsd1/FLT3-ITD leukemia. A. No recurrent Tier 1 somatic mutations were identified; one leukemia each had a mutation in Notch1 or Jak1. B. Loss of WT Flt3 allele in AML samples. Samples K731, K708, K693, and K704 have lost the WT Flt3 allele in the AML sample. M; 100 bp ladder.

## Slide 5
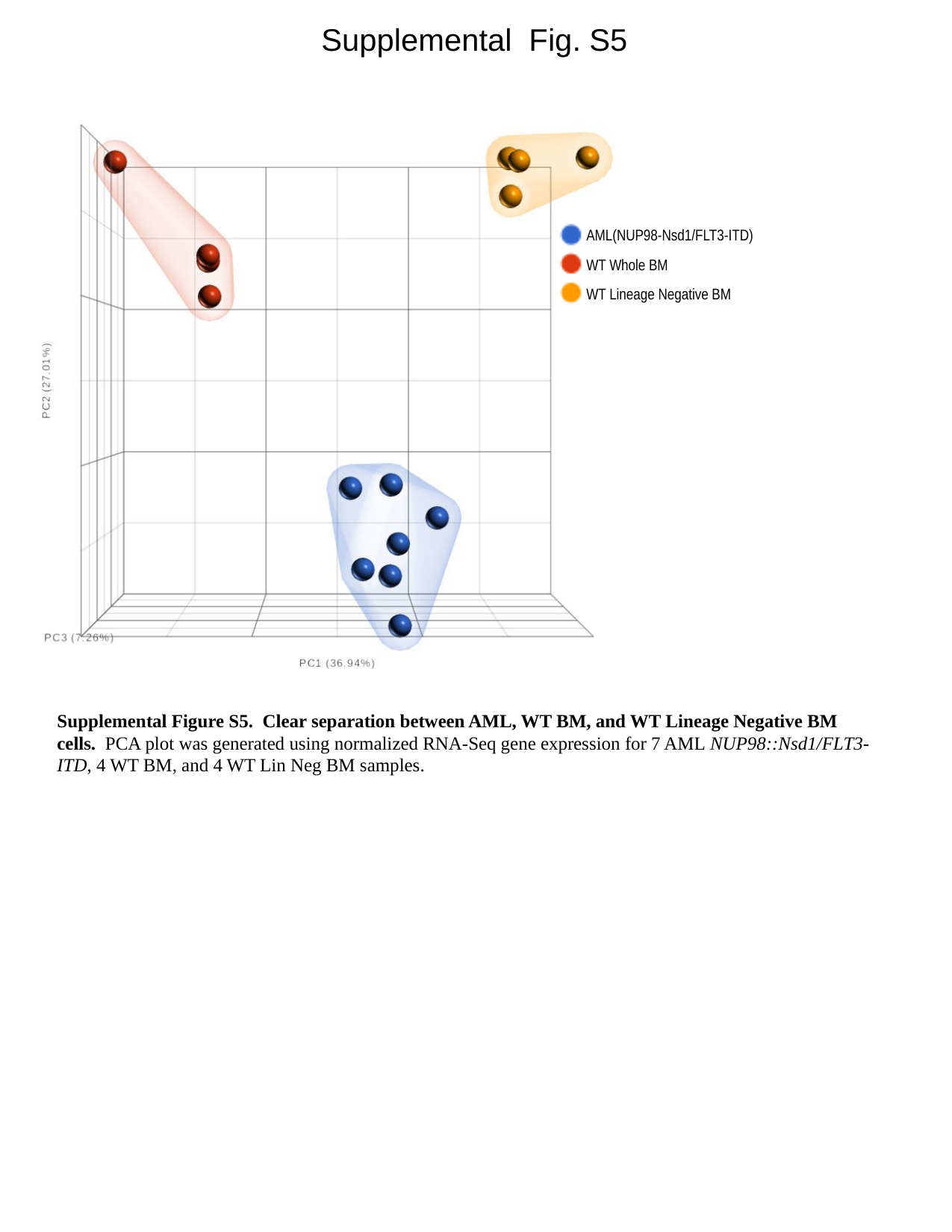

Supplemental Fig. S5
AML(NUP98-Nsd1/FLT3-ITD)
WT Whole BM
WT Lineage Negative BM
Supplemental Figure S5. Clear separation between AML, WT BM, and WT Lineage Negative BM cells. PCA plot was generated using normalized RNA-Seq gene expression for 7 AML NUP98::Nsd1/FLT3-ITD, 4 WT BM, and 4 WT Lin Neg BM samples.

## Slide 6
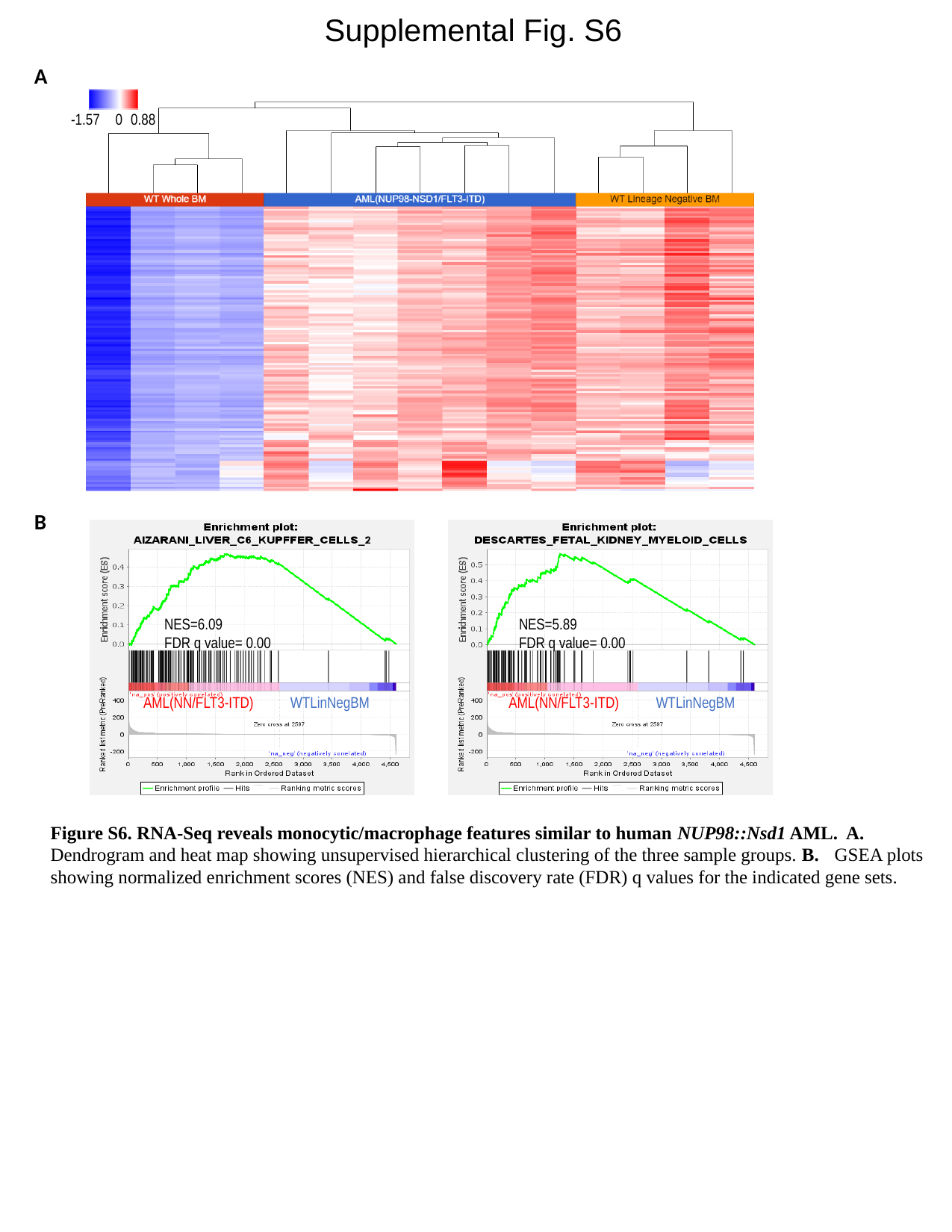

Supplemental Fig. S6
A
-1.57
0
0.88
B
NES=6.09
FDR q value= 0.00
WTLinNegBM
AML(NN/FLT3-ITD)
NES=5.89
FDR q value= 0.00
WTLinNegBM
AML(NN/FLT3-ITD)
Figure S6. RNA-Seq reveals monocytic/macrophage features similar to human NUP98::Nsd1 AML. A. Dendrogram and heat map showing unsupervised hierarchical clustering of the three sample groups. B.	GSEA plots showing normalized enrichment scores (NES) and false discovery rate (FDR) q values for the indicated gene sets.

## Slide 7
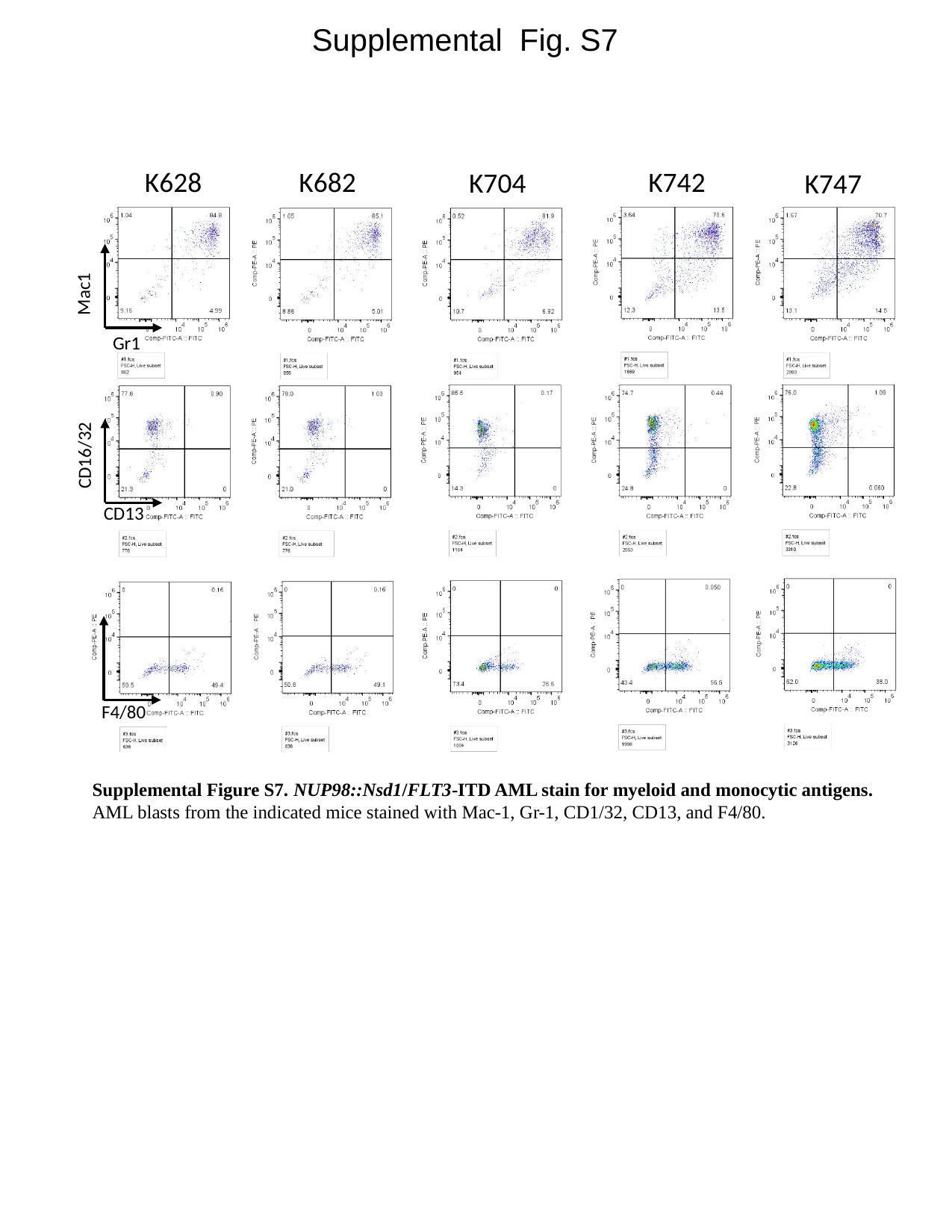

Supplemental Fig. S7
K682
K628
K742
K704
K747
Mac1
Gr1
CD16/32
CD13
F4/80
Supplemental Figure S7. NUP98::Nsd1/FLT3-ITD AML stain for myeloid and monocytic antigens.
AML blasts from the indicated mice stained with Mac-1, Gr-1, CD1/32, CD13, and F4/80.

## Slide 8
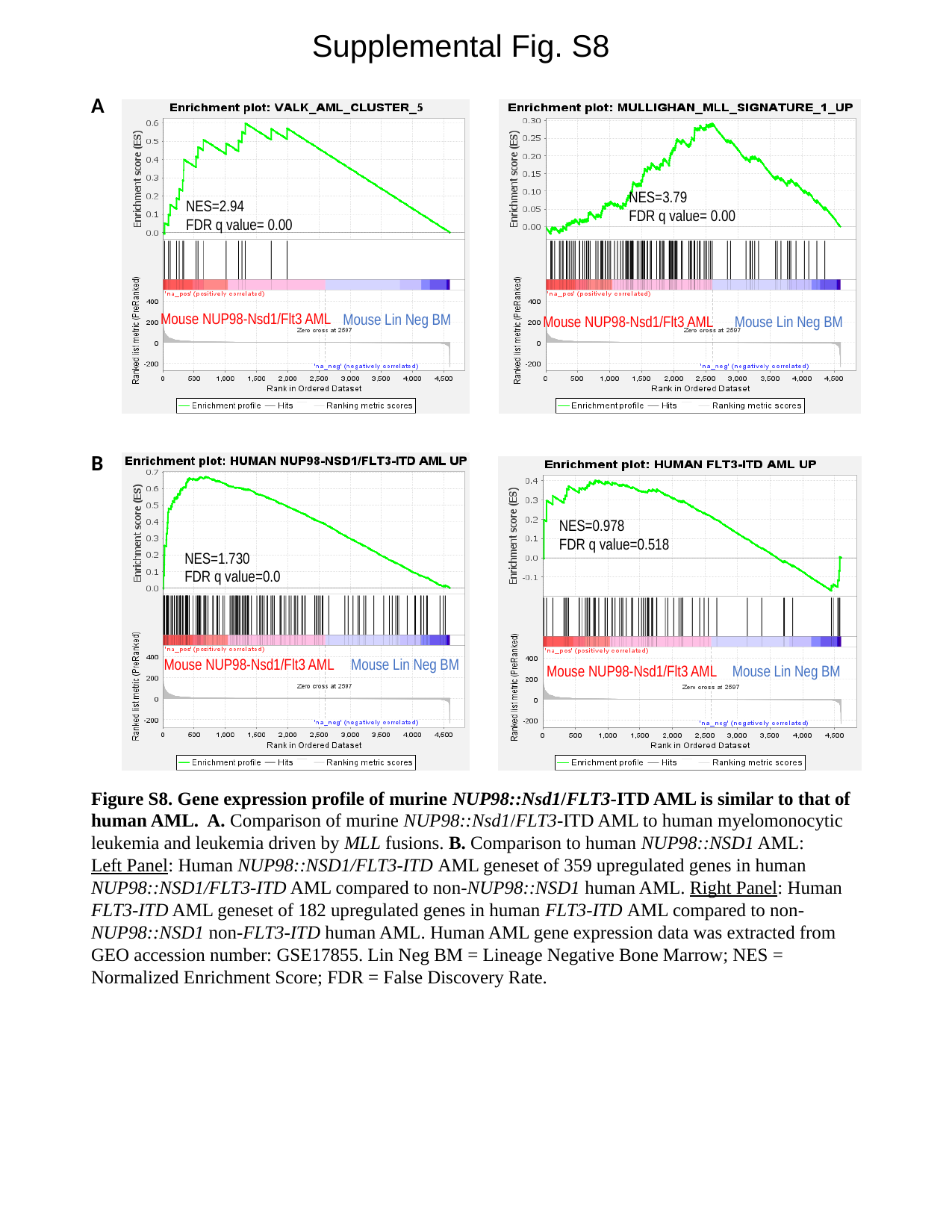

Supplemental Fig. S8
A
NES=3.79
FDR q value= 0.00
Mouse Lin Neg BM
Mouse NUP98-Nsd1/Flt3 AML
NES=2.94
FDR q value= 0.00
Mouse NUP98-Nsd1/Flt3 AML
Mouse Lin Neg BM
B
NES=1.730
FDR q value=0.0
Mouse Lin Neg BM
Mouse NUP98-Nsd1/Flt3 AML
NES=0.978
FDR q value=0.518
Mouse Lin Neg BM
Mouse NUP98-Nsd1/Flt3 AML
Figure S8. Gene expression profile of murine NUP98::Nsd1/FLT3-ITD AML is similar to that of human AML. A. Comparison of murine NUP98::Nsd1/FLT3-ITD AML to human myelomonocytic leukemia and leukemia driven by MLL fusions. B. Comparison to human NUP98::NSD1 AML:
Left Panel: Human NUP98::NSD1/FLT3-ITD AML geneset of 359 upregulated genes in human NUP98::NSD1/FLT3-ITD AML compared to non-NUP98::NSD1 human AML. Right Panel: Human FLT3-ITD AML geneset of 182 upregulated genes in human FLT3-ITD AML compared to non-NUP98::NSD1 non-FLT3-ITD human AML. Human AML gene expression data was extracted from GEO accession number: GSE17855. Lin Neg BM = Lineage Negative Bone Marrow; NES = Normalized Enrichment Score; FDR = False Discovery Rate.
